# Intrahepatic Cholangiocarcinoma Masquerading as Acute Fatty Liver of Pregnancy: A Case Report and Review of the Literature

**DOI:** 10.1155/2018/6939747

**Published:** 2018-02-26

**Authors:** Ayman Qasrawi, Omar Abughanimeh, Mouhanna Abu Ghanimeh, Simran Arora-Elder, Osama Yousef, Tarek Tamimi

**Affiliations:** ^1^Internal Medicine Department, University of Missouri-Kansas City School of Medicine, Kansas City, MO, USA; ^2^Internal Medicine Department, Division of Gastroenterology, Henry Ford Hospital, Detroit, MI, USA; ^3^Division of Hematology/Oncology, University of Maryland Medical Center, Baltimore, MD, USA; ^4^Division of Gastroenterology, University of Missouri-Kansas City School of Medicine, Kansas City, MO, USA

## Abstract

Cholangiocarcinoma (CCA) is an uncommon cancer and accounts only for 3% of all gastrointestinal malignancies. In this report, we present a case of an intrahepatic cholangiocarcinoma masquerading as acute fatty liver of pregnancy (AFLP). A 38-year-old female who is 36-week pregnant presented with a 1-week history of headache, nausea, vomiting, and right upper abdominal pain, along with hepatomegaly. Laboratory investigations were remarkable for mild leukocytosis, hyperbilirubinemia, proteinuria, and elevated transaminases and prothrombin time. Ultrasound of the liver revealed hepatomegaly, fatty infiltration, and a right hepatic lobe mass. Based on the overall picture, AFLP was suspected, and the patient underwent delivery by Cesarean section. However, bilirubin and liver enzyme levels gradually increased after delivery. MRI revealed a large dominant hepatic mass along with multiple satellite lesions in both lobes. Biopsy revealed the presence of intrahepatic CCA. CCA presenting during pregnancy is extremely rare with only 9 other cases reported in the literature. Therefore, the signs and symptoms can be easily confused with other more common disorders that occur during pregnancy.

## 1. Introduction

Cholangiocarcinoma (CCA) is an uncommon malignancy that arises from the epithelial cells of the biliary tree. CCA accounts for approximately 3% of all gastrointestinal malignancies [1, 2] and has a high mortality rate, given its late diagnosis and refractoriness to therapy [3]. On average, CCAs have a 5-year survival rate of 5–10% [4]. CCA presents extremely rarely during pregnancy and can mimic other disorders, such as obstructive cholestasis or HELLP syndrome [5, 6], which can lead to delayed diagnosis. We present a case of intrahepatic CCA that mimicked acute fatty liver of pregnancy (AFLP) and was subsequently diagnosed after delivery.

## 2. Case Presentation

**Figure 1:**
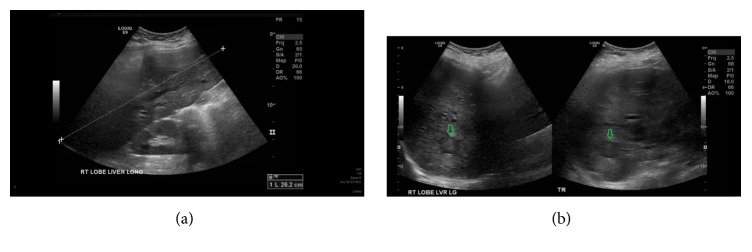
Liver ultrasound. (a) demonstrates the size of the liver measured to be about 26.2 cm. (b) demonstrates the hypoechoic mass in the right hepatic lobe (green arrows). The mass measured 2.8 cm.

**Figure 2:**
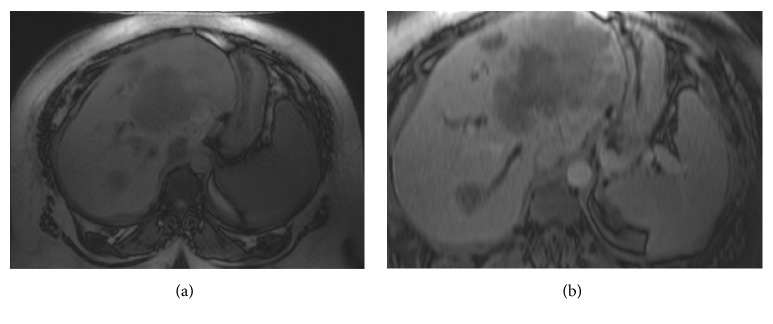
MRI of the liver and abdomen showing the large hepatic mass. (a) T1-weighted image. (b) T2-weighted image. The mass is involving the majority of the left hepatic lobe, measuring approximately 11.2 × 9.2 × 5.8 cm. There are multiple additional satellite lesions within the left hepatic lobe. There is an additional similar-appearing smaller T2 hyperintense lesion within the right hepatic lobe, measuring approximately 2.8 × 2.1 cm.

A 38-year-old female with morbid obesity and chronic hypertension presented in her fourth pregnancy at 36 weeks of gestation with a 1-week history of mild headache, nausea, epigastric and right upper quadrant pain, and dark urine. The pain started gradually but was constant and sharp in nature. Upon physical examination, she was jaundiced. The abdominal exam was remarkable for hepatomegaly and a gravid uterus. The neurological exam was normal. The patient denied use of any hepatotoxic medications. Her labs were completely normal about three weeks earlier. Initial laboratory workup showed a leucocyte count of 12.0 × 10^9^/L (70% neutrophils), platelet count of 450 × 10^9^/L, hemoglobin of 11.5 g/dL, total bilirubin of 6.4 mg/dl (direct fraction 5.0 mg/dL), aspartate aminotransferase (AST) of 83 U/L, alanine aminotransferase (ALT) of 87 U/L, alkaline phosphatase (ALP) of 319 U/l, glucose of 66 mg/dL, LDH of 679 U/L, uric acid of 4.2 mg/dL, and total serum bile acids of 71 *μ*mol/L (ref. 0–19 *μ*mol). Prothrombin time was 17 seconds with an international normalized ratio (INR) of 1.4. Viral hepatitis serology, autoimmune marker, and ceruloplasmin test results were unremarkable. The urine protein-to-creatinine ratio was elevated with 24 hours of collection for urine protein, 1300 mg/day; her 24-hour urine protein was 180 mg/day prior to pregnancy. Ultrasonography showed marked hepatomegaly (~27 cm), fatty infiltration of the liver, and a right hepatic hypoechoic 2.8 cm mass (Figure 1). Moreover, the liver exhibited heterogenous echotexture along with areas of nodular contour. Given the patients' clinical presentation, biochemical profile, and imaging findings, the obstetricians suspected AFLP. The patient underwent delivery by Cesarean section; however, bilirubin and liver enzyme levels gradually increased after delivery. Magnetic resonance imaging (MRI) of the liver was obtained three days after delivery for further evaluation of the mass and worsening liver function (Figure 2). MRI showed a heterogeneous T2 hyperintense mass involving the majority of the left hepatic lobe, measuring approximately 11.2 × 9.2 × 5.8 cm. There was an additional similar, smaller lesion within the right hepatic lobe, measuring approximately 2.8 × 2.1 cm. There were additional satellite lesions within the left hepatic lobe. There was mild, diffuse intrahepatic biliary dilation. Serum tumor markers were obtained and showed elevated cancer antigen 19-9 (CA19-9) of >10,000 U/ml, CEA of 160.5 ng/mL (normal up to 5.20), and *α*-fetoprotein (AFP) of 1,135 ng/mL. US guided biopsy tissue obtained from the smaller right hepatic mass showed adenocarcinoma with an immunohistochemical profile consistent with cholangiocarcinoma. A CT scan of the chest showed multiple bilateral pulmonary nodules suspicious for metastasis. Total bilirubin started to increase gradually, up to 14.0 mg/dL. The patient underwent placement of percutaneous biliary drains with subsequent improvement of her total bilirubin to 4.0 mg/dL. She was then started on palliative chemotherapy with gemcitabine; however, this was complicated by recurrent episodes of cholangitis with multidrug-resistant organisms. She also developed progressive disease and peritoneal carcinomatosis and was subsequently transitioned into hospice care. She died around six months after her original presentation.

## 3. Discussion

CCAs can be classified based on their anatomical location, as intrahepatic, perihilar, or distal extrahepatic [3]. The majority of CCAs are either perihilar or distal, with intrahepatic disease responsible for <10% of the cases [2, 7]. CCA risk factors include, but are not limited to, primary sclerosing cholangitis, choledocholithiasis, long-standing ulcerative colitis, infestation with* Clonorchis sinensis*, Caroli's disease, and congenital hepatic fibrosis [2, 3, 8]. In most patients, there is no identifiable cause of CCA [2]. The clinical features of CCAs differ according to their clinical location [2, 4]. In general, they are asymptomatic in the early stages and symptomatic cases usually indicate advanced disease [2, 3]. Extrahepatic tumors usually present with painless jaundice from biliary obstruction [2, 4]; on the other hand, intrahepatic CCAs are less likely to cause jaundice. Common CCA symptoms include abdominal pain, fatigue, cachexia and/or fever, and night sweats [2–4]. Intrahepatic CCAs can be an incidental finding, when imaging is obtained, as part of the workup of abnormal liver blood tests [9].

Liver diseases complicate the courses of ~3* *% of all pregnancies and some of them can have severe consequences [9–11]. They often have very similar presentations. The most important pregnancy-specific presentations are preeclampsia, eclampsia, hyperemesis gravidarum, AFLP, intrahepatic cholestasis of pregnancy, and HELLP syndrome [11, 12]. Additionally, the differential diagnosis includes other disorders that are unrelated to pregnancy, such as drugs, toxins, and viral hepatitis. AFLP is a rare but life-threatening disease that occurs mostly in the third trimester [11]. The usual symptoms of AFLP are nausea, vomiting, and epigastric pain [11, 12] and the notable laboratory features include leukocytosis, moderate elevation in liver enzymes, hyperbilirubinemia, coagulopathy, hyperuricemia, hypoglycemia, and proteinuria [11–13]. Ultrasound features include increased echogenicity, indicating fatty infiltration and sometimes ascites [11, 14]. Swansea criteria can also be used to aid the diagnosis [11].

The majority of liver masses identified during pregnancy are more commonly benign [15], for example, hemangiomas, adenomas, hamartomas, and focal nodular hyperplasia [15, 16]. Only a few cases of hepatocellular carcinoma were reported in the literature [15]; given the rarity of malignant liver lesions in pregnancy and that the presenting symptoms of malignancy may be confused with the common symptoms of pregnancy, the diagnosis is often delayed [5, 15]. In addition, diagnostic and interventional modalities are limited in pregnancy, which might be another limiting factor in early diagnosis [16].

CCA is extremely rare during pregnancy. We searched the PubMed database and found 9 cases in 8 reports of CCA in pregnancy from 1975 to 2015 [5, 6, 17–22]. We analyzed the 9 cases and the present case. The age of the women ranged from 25 to 38. Five of the cases were diagnosed in the second trimester and one in the third. In our case and in the case reported by Zelissen et al., the symptoms started in the pregnancy but the correct diagnosis was established postpartum [18]. In one of the cases reported by Purtilo et al., the patient died from meningitis during pregnancy and was found to have incidental CCA during autopsy [17]. In the other case reported by Purtilo et al., a postpartum woman had a positive pregnancy test and a metastatic malignancy. The diagnosis was confused with choriocarcinoma due to ectopic secretion of human chorionic gonadotropin; the correct diagnosis was established during autopsy [17]. Common presenting symptoms and signs were nausea, vomiting, abdominal pain, pruritus, jaundice, hepatomegaly, and/or a palpable mass; interestingly, one of the cases presented as spinal cord compression [21]. In the cases with reported laboratory values, the liver enzymes (AST/ALT) were normal or slightly elevated. Total bilirubin was elevated in five cases (range: 3.6–15.9 mg/dL). Other abnormal lab results were malignant hypercalcemia in one case and elevated bile acids in another [5, 18]. The diagnosis mimicked obstetric cholestasis in one case [5], HELLP in another [6], and acute fatty liver of pregnancy in our case. The prognosis was generally poor: six of the women died shortly, up to 6 months, after diagnosis. Pregnancy may adversely affect the prognosis of hepatocellular carcinoma, as gestational immune suppression may be an enabling factor in tumor progression [23]. This might be also true for CCA but cannot be proven due to the paucity of reported cases.

In conclusion, CCA presenting during pregnancy is extremely rare; however, the signs and symptoms can be easily confused with other more common disorders that occur in pregnant women. In addition, pregnancy might limit the diagnostic modalities, which can lead to delayed diagnosis and potentially worse outcomes.
